# Computable phenotype for real-world, data-driven retrospective identification of relapse in ANCA-associated vasculitis

**DOI:** 10.1136/rmdopen-2023-003962

**Published:** 2024-04-30

**Authors:** Jennifer Scott, Arthur White, Cathal Walsh, Louis Aslett, Matthew A Rutherford, James Ng, Conor Judge, Kuruvilla Sebastian, Sorcha O’Brien, John Kelleher, Julie Power, Niall Conlon, Sarah M Moran, Raashid Ahmed Luqmani, Peter A Merkel, Vladimir Tesar, Zdenka Hruskova, Mark A Little

**Affiliations:** 1 Trinity Kidney Centre, Trinity Translational Medicine Institute, Trinity College Dublin, Dublin, Ireland; 2 School of Computer Science and Statistics, Trinity College Dublin, Dublin, Ireland; 3 ADAPT SFI centre, Trinity College Dublin, Dublin, Ireland; 4 Department of Computer Science and Statistics, Trinity College Dublin, Dublin, Ireland; 5 National Centre for Pharmacoeconomics, St James's Hospital, Dublin, Ireland; 6 Department of Mathematical Science, University of Durham, Durham, UK; 7 School of Infection & Immunity, University of Glasgow, Glasgow, UK; 8 School of Medicine, College of Medicine, Nursing and Health Science, University of Galway, Galway, Ireland; 9 Department of Statistics, Dublin Institute of Technology, Dublin, Ireland; 10 Vasculitis Ireland Awareness, Dublin, Ireland; 11 Department of Immunology, St James's Hospital, Dublin, Ireland; 12 Department of Nephrology, Cork University Hospital, Cork, Ireland; 13 Nuffield Department of Orthopaedics, Rheumatology and Musculoskeletal Science (NDORMs), University of Oxford, Oxford, UK; 14 Division of Rheumatology, Department of Medicine, Division of Epidemiology, Department of Biostatistics, Epidemiology, and Informatics, University of Pennsylvania, Philadelphia, Pennsylvania, USA; 15 Department of Nephrology, General University Hospital, Prague, Czech Republic; 16 1st Faculty of Medicine, Charles University, Prague, Czech Republic; 17 General University Hospital, Prague, Czech Republic

**Keywords:** Vasculitis, Outcome Assessment, Health Care, Classification, Epidemiology

## Abstract

**Objective:**

ANCA-associated vasculitis (AAV) is a relapsing-remitting disease, resulting in incremental tissue injury. The gold-standard relapse definition (Birmingham Vasculitis Activity Score, BVAS>0) is often missing or inaccurate in registry settings, leading to errors in ascertainment of this key outcome. We sought to create a computable phenotype (CP) to automate retrospective identification of relapse using real-world data in the research setting.

**Methods:**

We studied 536 patients with AAV and >6 months follow-up recruited to the Rare Kidney Disease registry (a national longitudinal, multicentre cohort study). We followed five steps: (1) independent encounter adjudication using primary medical records to assign the ground truth, (2) selection of data elements (DEs), (3) CP development using multilevel regression modelling, (4) internal validation and (5) development of additional models to handle missingness. Cut-points were determined by maximising the F1-score. We developed a web application for CP implementation, which outputs an individualised probability of relapse.

**Results:**

Development and validation datasets comprised 1209 and 377 encounters, respectively. After classifying encounters with diagnostic histopathology as relapse, we identified five key DEs; DE1: change in ANCA level, DE2: suggestive blood/urine tests, DE3: suggestive imaging, DE4: immunosuppression status, DE5: immunosuppression change. F1-score, sensitivity and specificity were 0.85 (95% CI 0.77 to 0.92), 0.89 (95% CI 0.80 to 0.99) and 0.96 (95% CI 0.93 to 0.99), respectively. Where DE5 was missing, DE2 plus either DE1/DE3 were required to match the accuracy of BVAS.

**Conclusions:**

This CP accurately quantifies the individualised probability of relapse in AAV retrospectively, using objective, readily accessible registry data. This framework could be leveraged for other outcomes and relapsing diseases.

**WHAT IS ALREADY KNOWN ON THIS TOPIC**

- Relapse in clinical trials is defined using the Birmingham Vasculitis Activity Score>0. However, this metric is often missing or incorrectly scored in real-world data, resulting in inaccurate ascertainment of this key outcome.
- ‘Computable phenotypes’ (electronic algorithms) are used in electronic health records to automate the identification of patient subgroups and outcomes.

**WHAT THIS STUDY ADDS**

- This is the first study to demonstrate the feasibility of a pragmatic data-driven algorithm to accurately automate the identification of relapse, in real-world data.

**HOW THIS STUDY MIGHT AFFECT RESEARCH, PRACTICE OR POLICY**

- Our algorithm could be used by researchers to uniformly label relapse events in their registry, hence ensuring more accurate outcome ascertainment.
- Therefore, this study has the potential to increase the sample size of observational studies exploring relapse, which is a critical enabler for rare disease research.
- This framework could serve as an exemplar for other relapsing-remitting diseases and for automating the identification of other key outcomes or cohorts in registry data.

## Introduction

ANCA-associated vasculitis (AAV) is a relapsing-remitting autoimmune disease, resulting in incremental tissue injury. With the availability of highly effective agents to induce remission, maintenance of remission has emerged as a key research focus. The risk of relapse without prolonged continuous immunosuppression (IS) has remained relatively unchanged.^1 2^ Relapses result in cumulative disease-related and treatment-related damage,^3 4^ including a ninefold increased risk of end-stage kidney disease (ESKD) following renal relapse.^5^ However, maintenance IS to reduce relapse risk is expensive and not without risk of toxicity.^6^ Therefore, there is a pressing need for effective prediction models enabling personalised therapy, balancing extended use of immunosuppressive (IS) medications against relapse prevention. To build such models, we must first be able to label the relapse outcome accurately and uniformly.

The internationally adopted definition of relapse of AAV in clinical trials uses the Birmingham Vasculitis Activity Score (BVAS), with a rise in BVAS after attaining remission indicating relapse.^7^ However, in rea-world data, this metric is often missing or incorrectly scored; for example, relapse mimics and chronic damage may be scored as active vasculitis, resulting in false positives. Real-time BVAS scoring is challenging, as the clinical assessment may not be interpreted in the context of other factors such as trends in objective laboratory data and medications. Relapse can only truly be determined retrospectively. Indeed, in the clinical trial setting, BVAS assessment is often validated post hoc by an adjudication committee, considering the totality of clinical evidence available and with knowledge of subsequent events. These limitations of the gold-standard definition (BVAS>0) are acknowledged by the vasculitis community, resulting in non-standardised amendments, potentially hindering comparisons between studies.^8^ Increasingly, to maximise specificity, the requirement for ‘escalation in IS therapy’ in response to new/worsening active vasculitis is a fundamental component of the modified definition.^9–12^

Currently, in the Irish national registry, the probability of relapse for each patient encounter is determined by an expert adjudication committee. This was implemented to (a) increase sample size where BVAS was missing and (b) maximise the accuracy of our analyses, where BVAS was recorded incorrectly. This process is time-intensive and labour-intensive so we sought to automate this process and make the expert consensus process more transparent and accessible to other researchers.

A similar approach of creating ‘computable phenotypes’ (CPs) to automate identification of patient subgroups and outcomes, using a combination of data elements (DEs) (eg, billing, diagnostic or procedural codes, medications, laboratory tests), has been employed in electronic health records (EHRs) and claims data.^13 14^ This has been guided by the National Institutes of Health (NIH) Collaboratory^14^ in the context of pragmatic trials. The syntax defining these CPs supports programmatic medical phenotyping, without the need for expert human involvement, thereby operationalising disease concepts. This reliable, reproducible and valid process supports replicable queries of observational data across multiple sites.^14^ A set of CPs were developed for purposes of case-finding in AAV through EHRs; however, they do not include assessment of outcomes or disease state.^15^ We aimed to apply this paradigm to automate outcome ascertainment in a rare disease registry.

We present the development, interval validation and evaluation of a pragmatic data-driven algorithm to automate retrospective identification of relapse in AAV. Reproducible, reliable ascertainment of relapse in observational data, using objective readily available data, is critical to facilitating large-scale real-world analysis. Importantly, our algorithm does not predict future relapse, but rather defines the characteristics of a discrete event, and hence classifies relapse.

## Patients and methods

### Study participants

#### Rare kidney disease registry

The rare kidney disease registry and biobank, established in 2012, is a national, longitudinal, multicentre, cohort study.^16^ Although patients are recruited from renal, rheumatology and immunology centres, the registry is nephrology focused. Central storage of anonymised registry data is hosted on a secure password-protected web-based software platform, REDCap,^17 18^ hosted at Trinity College Dublin.

#### Longitudinal cohort

Patients were included if they were diagnosed with definite AAV^16^ at least 6 months beforehand and classified using the European Medicines Agency algorithm.^19^ Patients with secondary vasculitis and/or antiglomerular basement membrane disease were excluded.^19^ Participants were required to have at least one adjudicated encounter by 14 November 2022 (online supplemental figure 1). Only encounters >6 months from diagnosis were included to exclude possible primary treatment failures, as distinct from relapse.^20^

10.1136/rmdopen-2023-003962.supp1 Supplementary data

### Data description

Data used for model development^16^ ares detailed in step 2 (below), and further described in online supplemental methods.

### Steps in building the CP for relapse

#### Step 1: independent expert adjudication of encounters to assign the reference probability of relapse (ground truth)

The primary outcome was relapse, defined as the return of symptoms and/or signs of active vasculitis, supported by linked laboratory, radiological or histopathological evidence, the therapeutic decision at the time of the encounter and the clinical response to same. Encounters were adjudicated by a committee of expert clinicians in advance of the study (described further in online supplemental methods), using the patient’s entire medical records, to determine the reference ‘ground truth’—a process endorsed by the NIH.^14^ Where this was recorded, we evaluated the performance of the gold standard relapse definition (BVAS>0) against this ground truth ‘adjudicated probability of relapse’.

#### Step 2: selection of DEs and corresponding value sets

The optimal approach to model development employs a small a priori set of candidate items^21^ (discussed further in online supplemental methods). Therefore, a small number of DEs were selected using expert domain knowledge (elicited in a semiformalised approach, further discussed in online supplemental methods) and relevant literature, with a consideration for likely real-world data availability. All data were obtained during routine clinical care, using locally available laboratory and radiological testing methods. A cross-tabulation was performed between all variables and the squared scaled generalised variance-inflation factors (GVIFs)^22^ were calculated to assess for multicollinearity.

#### Step 3: development of a CP with an embedded logistic multilevel model

**Figure 1:**
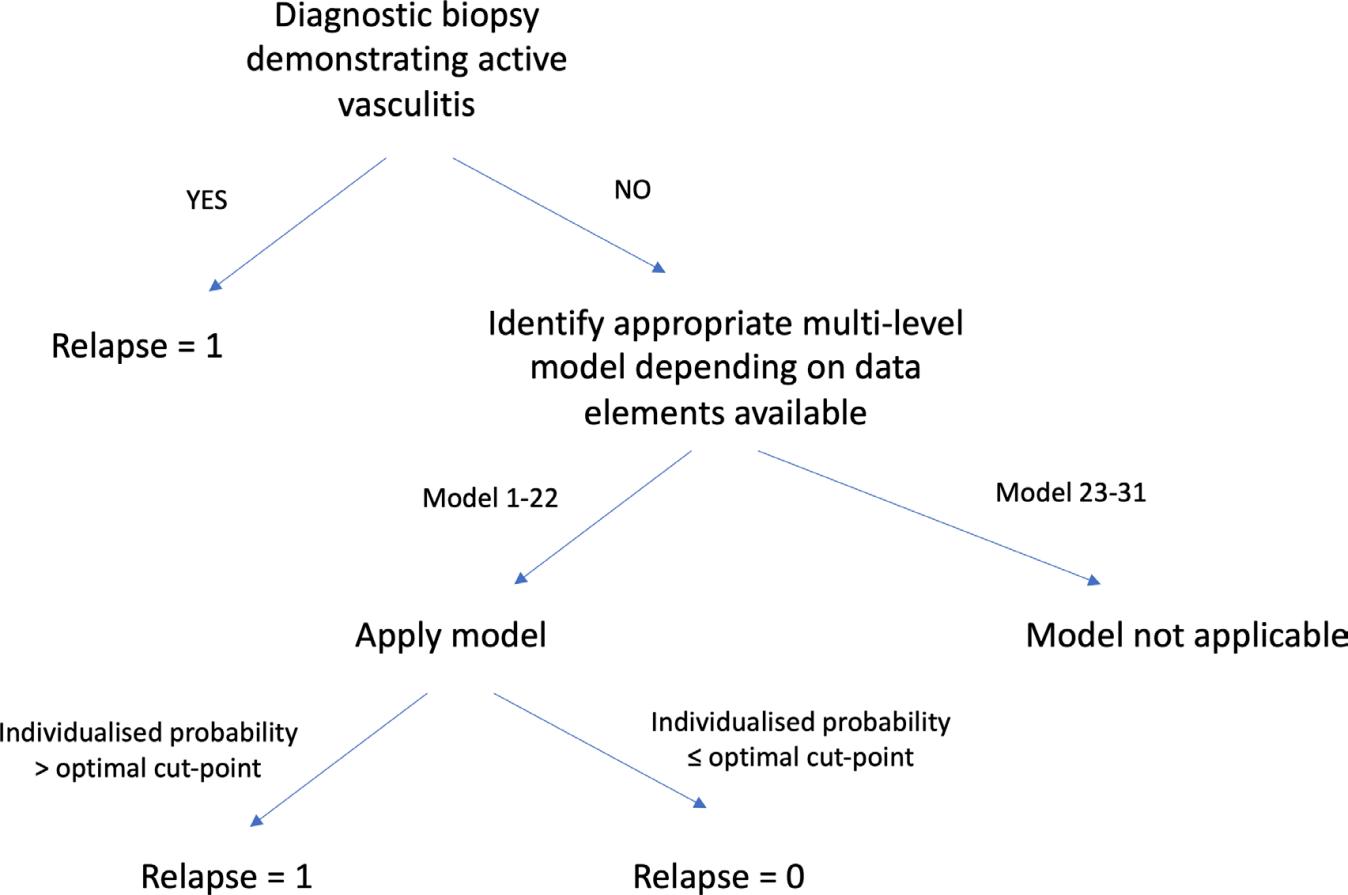
Proposed algorithm to define the computable phenotype for relapse. To identify the appropriate model for the corresponding data elements available refer to online supplemental table 3. The F1-score of models 23–31 was <0.70 (the point estimate of the F1-score of the BVAS>0 relapse definition), and therefore, they were deemed not applicable. BVAS, Birmingham Vasculitis Activity Score.

We considered that diagnostic histopathology demonstrating active vasculitis, in a patient previously in remission, equates to biopsy-proven relapse. It is the most objective gold-standard evidence available, but few patients undergo invasive biopsy. This logic was applied as the initial step in the algorithm (figure 1). For encounters without diagnostic histopathology, the five categorical DEs identified in step 2 were used as covariates to develop a logistic multilevel model (lme4 package(V.1.1–31), glmer function).^23^ This model was chosen (over a traditional cox proportional hazards model) as our aim was to define the characteristics of a discrete relapse event, agnostic to when it occurred, rather than model the probability of relapse over time (ie, it is a classification rather than a temporal prediction problem). Complete-case analysis was used as data were missing not-at-random. We used the ‘Transparent Reporting of a multivariable prediction model for Individual Prognosis Or Diagnosis’ (TRIPOD) statement^24^ to guide the development and internal validation of our model. The cohort was randomly split into development (80%) and validation (20%) sets at the patient level, with a similar proportion of relapses in both. A random effect was included to account for repeated encounters per patient and the varying relapse occurrence between individuals.^25^ The a priori risk of overfitting was deemed minimal given the small number of independent variables selected in advance. ORs and 95% CIs were computed. A p<0.05 was considered statistically significant. All statistical analyses were performed by using R V.4.2.1.

#### Step 4: internal validation

Model discrimination was assessed with the following metrics: F1-score (the harmonic mean of recall (equivalent to sensitivity) and precision (equivalent to positive predictive value (PPV)), whereby F1-Score=2×(precision×recall)/(precision+recall)),^26^ sensitivity, specificity, PPV, negative predictive value (NPV), accuracy and area under the receiver operating characteristic (ROC) curve^27^ (AUC, whereby 1.0 represents ideal discrimination and 0.5 indicates discrimination that is no better than chance). The optimal cut-point was determined by maximising the F1-Score (R package: cutpointr V.1.1.2), as appropriate in an imbalanced dataset,^26^ where it can be viewed akin to classification accuracy. This was also chosen based on the proposed use case of determining the true relapse rate in the cohort, where balancing sensitivity (correctly identifying relapse when it exists) with precision (minimising false positives) is important.^28^ The multilevel logistic regression analysis was reiterated fifty times using stratified random-split resampling^29^ to ensure stability and reproducibility of the model^14^ and to control for potential sample-specific overfitting, hence minimising type-1 error. In each resampling trial, the model was constructed using a randomly chosen portion of 80% of the data (development set) and the predictive accuracy was tested in the remaining 20% (validation set) by calculating the performance metrics listed above. The mean and 95% CI of the sampling distribution of each performance metric were computed across the fifty iterations. Calibration was assessed by comparing the estimated probabilities of the outcome and the observed outcome’s proportion.^27^ We performed prespecified subgroup analysis stratified by kidney involvement.

#### Step 5: model development when variables are missing

Steps 3 and 4 were repeated for each of the remaining thirty combinations of five DEs to determine if and when a stable model could be developed in the case of one or more missing DEs. We then developed an R shiny web application (https://jennifer-scott.shinyapps.io/Relapse_identification/) for use by researchers wishing to apply the CP to their data. This application automatically applies one of the 31 possible models, based on available data, to generate an individualised probability of relapse and the corresponding binary label (determined by the individually determined optimal cut-point) for each observation. The overall classification accuracy of each model was ranked by maximal F1-score. The 95% CI of the F1-scores was used to compare the F1-score between models. Models were considered suitable if the 95% CI of the F1-score crossed 0.7 (the F1-score point estimate when the ‘BVAS>0’ classification was used as the relapse label). ‘Not applicable’ is returned by the web application for observations where the corresponding model did not meet this criterion. A second internal validation was performed using this web application on the incomplete cases (ie, those excluded from complete-case analysis).

### Patient and public involvement

Patient and public involvement is detailed in online supplemental methods.

## Results

### Participant characteristics

536 patients met the inclusion criteria (online supplemental figure 1), with 3387 adjudicated encounters over a median follow-up of 72 months (table 1 details their characteristics). 58% were male, predominantly (99%) White, with a median age of 60 years at diagnosis. 40% experienced at least one relapse and 13% died during follow-up.

**Table 1:** Baseline patient characteristics in study cohort overall and stratified by complete case analysis, split into train and test sets

Variable	Overall* (complete and Incomplete cases)	Total complete cases	Train	Test
n	536	416	334	82
Age at diagnosis (years, median (IQR))	60.0 (49.0–69.0)	59.0 (48.0–69.0)	61.0 (48.3–69.0)	56.0 (48.0–63.5)
Male (N (%))	310 (57.8)	236 (56.7)	191 (57.2)	45 (54.9)
Race (N (%))				
White	529 (98.7)	410 (98.6)	329 (98.5)	81 (98.8)
Asian	7 (1.3)	6 (1.4)	5 (1.5)	1 (1.2)
AAV Phenotype (N (%))				
Granulomatosis with polyangiitis (GPA)	261 (48.7)	205 (49.3)	168 (50.3)	37 (45.1)
Microscopic polyangiitis	249 (46.5)	189 (45.4)	148 (44.3)	41 (50.0)
Eosinophilic GPA (EGPA)	26 (4.9)	22 (5.3)	18 (5.4)	4 (4.9)
ANCA serotype (N (%))				
Proteinase-3 (PR3)	276 (51.5)	220 (52.9)	175 (52.4)	45 (54.9)
Myeloperoxidase (MPO)	243 (45.3)	182 (43.8)	149 (44.6)	33 (40.2)
MPO and PR3	3 (0.6)	3 (0.7)	1 (0.3)	2 (2.4)
ELISA negative	13 (2.4)	10 (2.4)	8 (2.4)	2 (2.4)
No ELISA performed	1 (0.2)	1 (0.2)	1 (0.3)	0 (0.0)
Organ involvement (N (%))				
Kidney	450 (84.0)	347 (83.4)	283 (84.7)	64 (78.0)
Lung	277 (51.7)	218 (52.4)	180 (53.9)	38 (46.3)
Musculoskeletal	209 (39.0)	163 (39.2)	130 (38.9)	33 (40.2)
Ear, nose and throat	238 (44.4)	185 (44.5)	148 (44.3)	37 (45.1)
Mucocutaneous	128 (23.9)	106 (25.5)	85 (25.4)	21 (25.6)
Neurologic	75 (14.0)	56 (13.5)	42 (12.6)	14 (17.1)
Gastrointestinal	29 (5.4)	23 (5.5)	16 (4.8)	7 (8.5)
Cardiovascular	13 (2.4)	10 (2.4)	8 (2.4)	2 (2.4)
Induction treatment (N (%))				
Cyclophosphamide	332 (61.9)	255 (61.3)	207 (62.0)	48 (58.5)
Rituximab	103 (19.2)	82 (19.7)	64 (19.2)	18 (22.0)
Cyclophosphamide and rituximab	31 (5.8)	27 (6.5)	25 (7.5)	2 (2.4)
Other	70 (13.1)	52 (12.5)	38 (11.4)	14 (17.1)
Status (N (%))				
Alive	462 (86.2)	365 (87.7)	293 (87.7)	72 (87.8)
Dead	72 (13.4)	49 (11.8)	40 (12.0)	9 (11.0)
Lost to follow-up	2 (0.4)	2 (0.5)	1 (0.3)	1 (1.2)
End-stage kidney disease (N (%))	96 (17.9)	62 (14.9)	46 (13.8)	16 (19.5)
Ever relapsed during follow-up (N (%))	213 (39.7)	183 (44.0)	143 (42.8)†	40 (48.8)†
Follow-up period (months, median (IQR))	72.5 (39.9–139.0)	75.4 (42.1–144.8)	71.5 (41.3–144.3)	81.6 (50.5–147.5)

Number (N), IQR, ELISA.

*536 patients (3387 encounters, comprising complete and incomplete cases) were included in the overall study: 416 had ≥1 complete adjudicated encounters (1586 encounters), which underwent an 80/20% split into train (n=334, 1209 encounters) and test (n=82, 377 encounters) sets for model building, respectively. There were no significant differences between the characteristics of the train and test sets. The remaining 1801 encounters with ≥1 missing fields were used as a second internal validation test set (see online supplemental figure 1).

†Due to repeated encounters per individual, the prevalence of relapse was 17% in the train and test sets.

### Relapse defined by BVAS >0: a real-world evaluation

Of the adjudicated encounters, BVAS was available for 1066 (31% completion rate in our registry). This is consistent with the degree of missingness across six additional European vasculitis registries, in the FAIRVASC consortium^30^ (online supplemental figure 2A). When comparing the adjudicated probability of relapse (ground truth), the F1-Score of the BVAS entries was 0.70 (online supplemental figure 2B). Online supplemental figure 2C illustrates the degree of false positives and negatives.

### Selection of DEs and corresponding value sets for the proposed model

Table 2 details the chosen DEs with their corresponding categorical drop-down options (value sets). Value sets were explored and merged to eliminate levels with small counts that precluded model convergence. There was no collinearity between variables, with all GVIFs centring around 1. The frequency and combination of these DEs and value sets are represented graphically in online supplemental figure 3.

**Table 2:** New data elements (DEs) with their corresponding value set (ie, categorical drop-down options) applied to the registry to uniformly summarise patient encounters, with regards to relapse probability

DE key	DE	Value set (categorical values)
DE1	Change in ANCA level DE1 was established using serial direct ELISA or indirect immunofluorescence (IF, if ELISA data missing) results: The delta value (%) of the ANCA titre (anti-MPO or anti-PR3) was calculated between the encounter of interest and the preceding value, which was on average 3 months prior (maximal 12 months). Delta=(encounter of interest value–preceding value)/preceding value×100 (400%=4 fold rise) If ELISA results were missing, IF results were used to infer the delta value: negative->positive=‘<4-fold rise’ negative->negative=‘no rise’ positive->positive=‘no rise’ positive->negative=‘no rise’	4-fold rise <4 fold rise No rise
DE2	Suggestive bloods/urine A composite of at least one suggestive blood(s) and/or urine biomarker result(s) including the occurrence of at least one of the following: 20% rise in creatinine level, new haematuria (>10 red blood cells per high power field and/or >3+ blood on dipstick), new proteinuria (>3+ protein on dipstick), C reactive protein above upper limit of normal or 20% rise in urine soluble CD163 (usCD163, normalised to urine creatinine) to a value >400 ng/mmol (Euroimmun assay).	Suggestive of relapse Not suggestive
DE3	Suggestive imaging Suggestive imaging incorporated all modalities and was considered ‘suggestive of relapse’ if the radiologist reported a finding consistent with active vasculitis. Findings included, but were not limited to, new or worsening destructive nasal disease, pulmonary nodules or cavitating lesions. ‘Not suggestive’ was selected if imaging was performed but it did not show signs of active vasculitis.	Suggestive of relapse Not suggestive No imaging performed
DE4	Immunosuppression (IS) status DE4 summarises the IS therapy at the time of the encounter. ‘Currently on IS’ describes the ‘IS status’ of a patient on prednisolone >10 mg and/or an additional IS agent (eg, azathioprine) at the time of the encounter.	Currently on IS Discontinued within 6 months Discontinued >6 months
DE5	IS medication in response to the encounter (IS response) The change in IS by the physician, in response to their clinical assessment, was dichotomised into ‘IS increased’ or not in DE5. An increase was defined as: a clinically meaningful escalation in the dose of the same agent, as determined by the treating physician (as distinct from dose optimisation/titration when an agent is commenced). An increase in prednisolone dose to >20 mg was considered clinically significant. a switch from a typical maintenance agent (or regimen) to a typical induction agent (eg, azathioprine switched to cyclophosphamide or an induction rituximab regimen).	Increased Not increased

### Derivation of five-variable model performance

**Figure 2:**
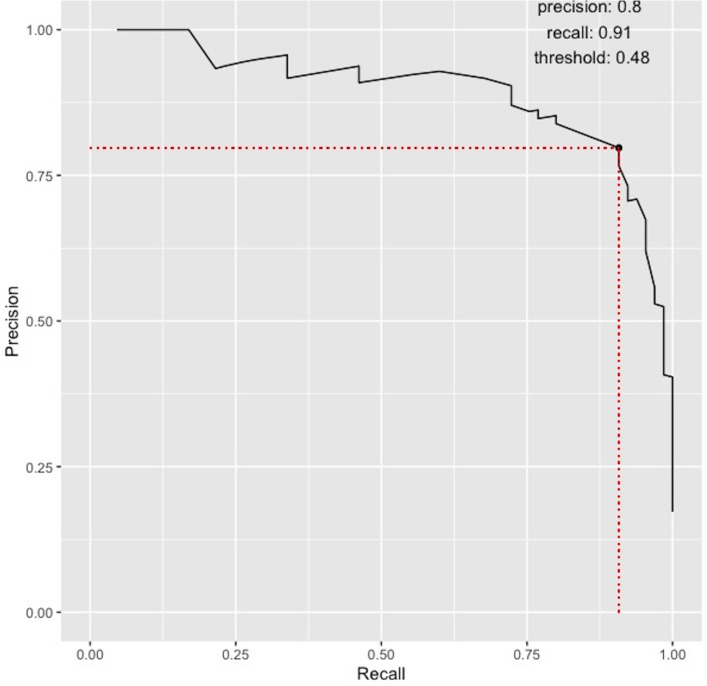
The precision-recall curve (PRC) of the complete five-variable model. The PRC is determined by plotting recall (sensitivity/true positive rate) against precision (positive predictive value). In the case of ‘rare’ events, such as relapse, a PRC is more appropriate than a receiver operating characteristic curve which can overestimate performance. A ‘perfect’ model is depicted by a PRC in the upper-right, passing the (1,1) coordinate. The optimal cut-point of 0.48 was determined by harmonising precision and recall, denoted by the maximal F1-score of 0.85.

1586 complete encounters across 416 unique patients were used for model building: 1209 encounters in the development set and 377 in the validation set. The prevalence of relapse was 17% in both. The OR (95% CI) of each DE using the complete five-variable model is reported in table 3, along with the performance metrics when applied to the validation set. The model’s high discriminative ability is visible on the precision-recall (figure 2) and ROC curves (AUC 0.98 (0.92–0.99), online supplemental figure 4). ‘Calibration-in-the-large’ was satisfied, whereby the observed rate of relapse (0.1724) was not statistically different to the average of all predicted probabilities (0.1707). Online supplemental figure 5 shows the calibration plot: the model is well calibrated at the extremes (close to 0/1), but there are insufficient data to assess calibration in between, which is akin to ‘possible’ cases in real-life clinical practice. Online supplemental figure 6 illustrates the performance of the model against the ground truth. The increased uncertainty regarding the diagnosis of minor relapses (eg, mild ear, nose and throat (ENT) or musculoskeletal symptoms) in clinical practice is reflected in the model (online supplemental figure 7). In subgroup analysis, the performance metrics stratified by kidney involvement are reported in online supplemental table 2. While the metrics are higher for those with kidney involvement, the F1-score is higher than 0.7 (the point estimate of the BVAS-derived definition) in both groups.

**Table 3:** Multilevel logistic regression model to identify the relative importance of exploratory variables in retrospective identification of relapse

	OR (95% CI)
No rise in ANCA level (Ref.)	–
<4 fold rise in ANCA level	**3.57 (1.34 to 9.50**)
4-fold rise in ANCA level	2.56 (0.96 to 6.83)
Bloods/urine not suggestive (ref.)	–
Suggestive bloods/urine	**5.59 (2.37 to 13.16**)
No imaging (ref.)	–
Imaging is not suggestive	2.38 (0.54 to 10.54)
Suggestive imaging	**30.20 (6.84 to 133.31**)
Currently on IS (ref.)	–
D/C of IS within 6 months prior	1.01 (0.27 to 3.78)
D/C of IS >6 months prior	1.87 (0.81 to 4.34)
IS not increased (ref.)	–
IS increased	**388.25 (102.68 to 1468.00**)
Optimal cut-point (max F1-score)	0.4789
F1-score	0.8489
Sensitivity	0.9077
Specificity	0.9519
Positive predictive value	0.7973
Negative predictive value	0.9802
Accuracy	0.9443
Area under ROC curve	0.9763
Prevalence of relapse	0.1724

Complete data, N=train 1209/test 377 (where N refers to the number of encounters). The Odds Ratios (ORs) (95% CI) are reported. The OR refers to the probability of the encounter being adjudicated as a relapse (relative to remission). The bold values highlight the variables that are significant in the model.

The performance metrics reported above are based on a singular train/test split (the corresponding cross-tabulation is reported in online supplemental table 1). The average metrics and 95% CI over 50 random-split resampling are reported in online supplemental table 3.

D/C, discontinuation; IS, immunosuppression; ROC, receiver operating characteristic curve.

### Model performance when DEs are missing

**Figure 3:**
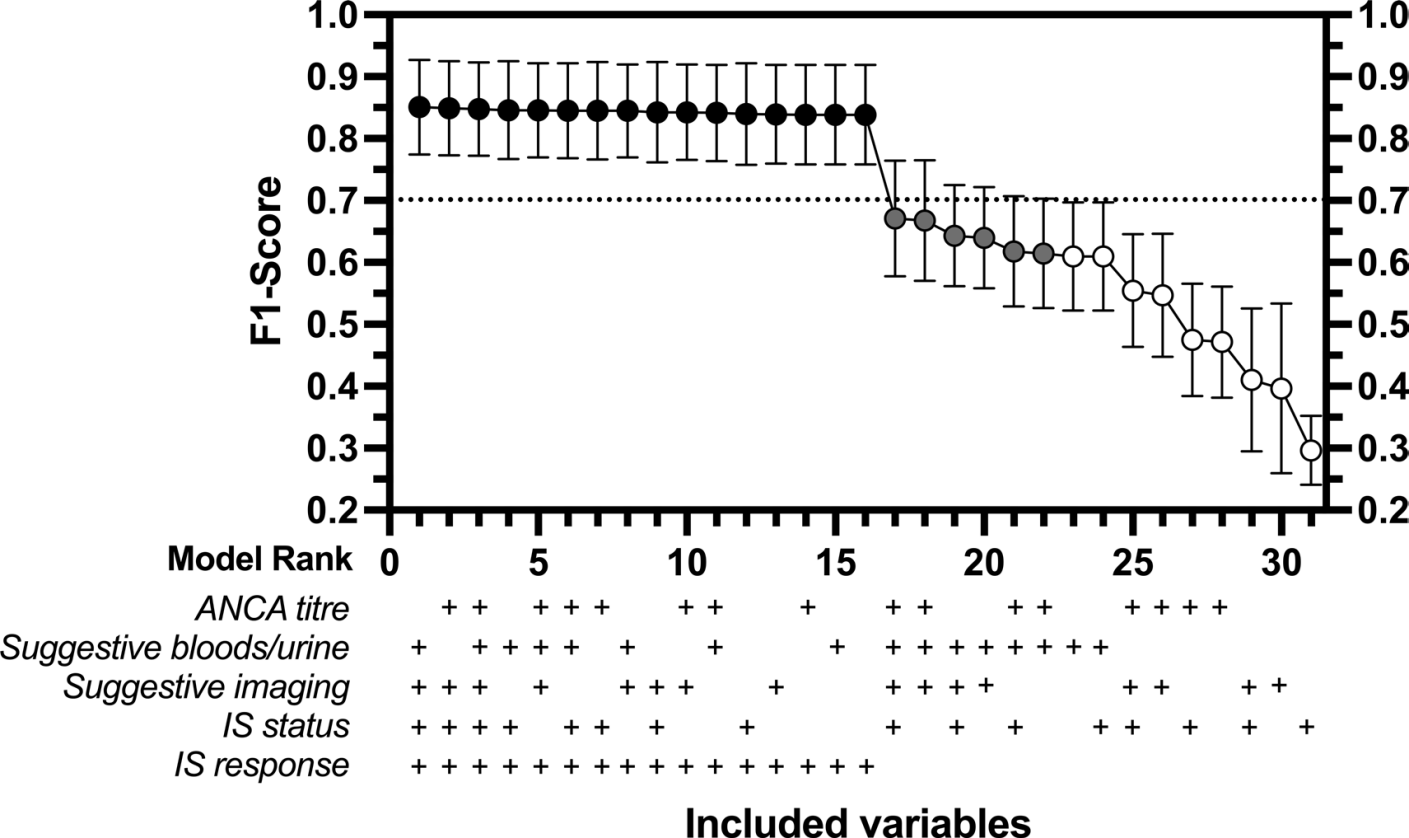
The mean of the F1-score and 95% CI for the 31 rank ordered models, to demonstrate the overall classification accuracy of the computable phenotype for relapse. Dotted line denotes F1-score for definition of relapse being BVAS>0 (0.70). Black dots (models 1–16) represent models with a classification accuracy superior to BVAS. The grey dots (models 17–22) denote models with similar performance to BVAS, and the white dots (models 23–31) represent models that are inferior to BVAS when comparing the F1-score as a marker of overall classification accuracy. The full performance metrics for each model rank are reported in online supplemental table 3. BVAS, Birmingham Vasculitis Activity Score; IS, immunosuppressive.

The models ranked according to F1-score are displayed in figure 3 and additional performance metrics (mean, 95% CI) are reported in online supplemental table 3. Models 1–16 include ‘IS response’(DE5) and are virtually identical in their classification accuracy. These models do not overlap the F1-score point estimate of the BVAS-derived definition (0.7), suggesting that models 1–16 have a superior classification accuracy in identifying relapse. The specificity of these models was very high (>0.95, approximately 10% higher than that of BVAS). The sensitivity was also high, ranging from 0.88 to 0.90, although lower than that of BVAS (0.97). Despite a similar prevalence of relapse in both samples, the PPV was much higher in models 1–16 (0.80–0.82) vs BVAS (0.55). In practice, this would equate to substantially fewer false positives while the NPV was unchanged (suggesting the false negative rate would be similar to BVAS). Models 17–22 (missing DE5, with at least DE2+DE1/3) performed similarly to BVAS, while models 23–31 (without at least DE2+DE1/3) had an inferior performance.

Applying the web interface we developed, we performed a second internal validation study using the 1801 incomplete encounters (initially excluded because of missing DEs, online supplemental figure 1). The performance metrics remained high (online supplemental figure 8).

## Discussion

We have developed and validated a reproducible digital algorithm (a CP) to accurately identify relapse retrospectively using objective registry data. The discrimination and calibration of this CP is as good as the current gold standard BVAS>0 relapse definition, even when some DEs are missing. Implementation of this CP using our web application will enable reliable ascertainment of relapse in observational data, when BVAS is missing or inaccurate. This, in turn, will facilitate large-scale real-world analysis, including the accurate reporting of relapse rates and the development of relapse prediction models.

In recent years, the traditional relapse definition of BVAS>0 has sometimes been modified to include the requirement for ‘an escalation in IS therapy’.^9–12^ However, this expanded definition is still unhelpful when BVAS is missing (69% in our registry, despite focused data entry). We took the view that an increase in IS (in a patient with prior remission) is akin to the physician’s actionable response to increased vasculitis activity—in essence, a more specific BVAS proxy—negating the need for a unique DE to represent disease activity. Occasional patients had increased disease activity without an ‘IS escalation’, or the converse. We attempted to include ‘clinical response to an escalation in IS’ (ie, did signs/symptoms reduce in response to treatment?), but this registry field was often missing. Ultimately, in settings where BVAS may be incorrect or incomplete, an increase in IS (DE5) is a simple and objective identifiable action.

The gain in classification accuracy by combining additional medication, biomarker, imaging and biopsy DEs to this augmented BVAS definition (>0 with IS escalation) has not been tested. All of these DEs, and crucially their trajectories, factor into the expert’s decision-making process during adjudication of encounters. The IS status (DE4) of the patient at the time of the encounter was selected as those off maintenance therapy are at higher relapse risk.^31^ We further interrogated whether the duration off IS influenced the adjudicated relapse probability by creating a three-level value set: currently on, recently ceased (within 6 months) or discontinued IS for >6 months, but no clear signal was observed.

The remaining three DEs (DE1–3) summarise the objective evidence used by clinicians in determining whether a relapse occurred, with the aim of increasing the sensitivity and specificity of the CP. Radiology (DE3) is often useful in patients with ENT and respiratory involvement and may be the only objective evidence available in non-renal patients. Imaging is typically only performed when there is suspicion of active disease, and hence it is unsurprising that this DE is strongly predictive of relapse. We view this akin to a weighting on the physician’s assessment. DE 2 is a composite of at least one suggestive biomarker result(s) from a list of five key items. A composite was chosen to reflect real-world practice, where not all investigations are performed at each encounter. The definition of ‘new haematuria’ was in line with the BVAS criteria^7^ and the same cut-off for ‘new proteinuria’ was chosen for consistency. Although considered a non-specific marker of inflammation, when used in combination with other DEs, a C reactive protein value above the normal range provides additional objective evidence of immune activation.^32^ A ‘20% rise in usCD163 with a titre >400 ng/mmol’ was chosen based on prior work by our group.^33^ While we acknowledge usCD163 is not in widespread use currently, it is not required for DE2, although it provides additional information if available. It is possible, however, that the relative weight of DE2, and potentially the algorithm performance overall, may differ when one or more of the composite items (eg, usCD163) is not measured. Furthermore, automating the completion of these DEs removes the physician’s interpretation that ‘significant findings are attributable to active vasculitis’, resulting in a possibility of incorrect scoring (eg, haematuria due to menstruation). However, one isolated incorrect DE will not raise the outputted probability enough to give a false positive. The addition of these diagnostic tests to our proposed algorithm is in keeping with methods described in the EHR context, such as the addition of haemoglobin A1c or brain natriuretic peptide to diabetes^14^ or heart failure^34^ definitions, respectively.

The diagnostic and prognostic value of serial ANCA testing for relapse is controversial, with heterogeneity of multiple study variables,^35 36^ requiring pragmatic decisions in our study design. We chose a ‘fourfold rise in ANCA level’ a priori, as the summary metric, based on the largest systematic review at the time of design, demonstrating its association with an almost threefold rise in subsequent relapse.^37^ A fourfold rise in our assay also equates to a positive result at the time of potential relapse. Surprisingly, there was minimal difference in the effect between a ‘<4 fold rise in ANCA’ and a ‘>4 fold rise in ANCA’, suggesting an alternative metric to summarise the change in ANCA level may be more appropriate. In clinical practice, as in our study, the sampling interval (between ANCA measurements) varies. Our 12-month interval limit may be too broad; modelling the slope of the rise,^36^ reappearance of ANCA or negative-positive switch may be better. Identifying the ‘optimal’ summary metric for the ANCA trajectory is a current focus of our research group. We will explore exchanging the ‘fourfold rise’ with this identified parameter in future iterations of the CP, which may alter the magnitude of effect of this DE. It is also important to highlight that 84% of our cohort had renal involvement. The superior performance of the complete model in this subgroup is likely due to the higher predictive value of ANCA rise in those with renal involvement,^11^ and the presence of other useful renal biomarkers. External validation is required to assess the generalisability of our algorithm to non-renal cohorts. The optimal model(s) may differ for patients with non-renal disease in terms of relative weighting of DEs and indeed the specific DEs included.

CPs should leverage data that are routinely collected. All DEs and value sets are identical in the EUVAS model registry,^38^ which has been adopted by multiple countries. Most DEs are available in the encounter-based registries of the FAIRVASC initiative^30^ (online supplemental table 4). This initiative aims to link registries, to facilitate large-scale vasculitis research.

Missing data and variation in DEs across registries is a fundamental challenge in wide-scale implementation of CPs. Therefore, we explored the performance of all potential combinations of the five selected DEs. We propose that all models^1–22^ with an F1-score similar or superior to that of the F1-score point estimate for BVAS>0 (0.70) should be considered a reasonable alternative. While ‘IS response’(DE5) alone performs very well, the addition of other DEs increases specificity. Models 17–22, which include ‘suggestive bloods/urine’(DE2) with at least either ‘ANCA level’(DE1) or ‘suggestive imaging’(DE3), demonstrate the value of the other DEs in accurately assigning an ‘adjudicated probability of relapse’ in the absence of ‘IS response’. Our second internal validation is a proof of concept of our web application to programmatically apply the CP without requiring coding skills. The performance remains strong in this previously unseen cohort of incomplete encounters, further demonstrating the possibility of accurately assigning a relapse label even when the degree of missingness across DEs varies. When our CP is applied to other cohorts, the relative importance of the DEs may vary and we, therefore, encourage collection of all variables pending further validation. Once externally validated, we recommend adoption of the agreed standardised DEs across all vasculitis registries to support a consistent relapse algorithm regardless of location.^39^ This standardisation goal is supported by the National Institutes of Health (NIH) Common Data Element initiatives^40^ and the Value Set Authority Centre^41^ of the National Library of Medicine. Outcome Measures in Rheumatology supports the development of Core Outcome Sets, including data-driven outcome measures, for use in clinical research and is therefore a potential vehicle for widespread adoption of our proposed CP, once validated.

There is no consensus on the optimal performance metric on which to assess a model’s performance. Therefore, we report multiple discrimination metrics, as well as calibration, in keeping with guidelines.^24^ Similarly, the best cut-point on which to dichotomise the outcome is use-case dependent.^42 43^ We used the maximal F1-score (a harmonic mean of precision and recall) to determine the cut-point in our imbalanced dataset (relapse: no relapse occurs approximately 1:4).^26^ In our case, maximising both recall, otherwise known as sensitivity (to identify relapse cases when they exist, ie, minimising false negatives) and precision, otherwise known as PPV (minimising false positive cases) are equally important. In alternative scenarios, different trade-offs may be more appropriate. For example, in pharmacoepidemiological research, a relapsing cohort on which to test a new medication may be required and, therefore, specificity is prioritised to reduce the potential impact of misclassification on risk estimates.^43^ In our registry, we observed a high number of false positives when using the BVAS>0 definition to identify relapse, denoted by the low PPV (0.55). In practice, this equates to approximately 50% of cases being labelled as relapse when they were actually in remission. The number of false positives reduced substantially using our algorithm (PPV 0.80), with little effect of the NPV (ie, the number of false negatives, or ‘missed’ relapses). So, while false positives are still greater than false negatives when using our algorithm, they are substantially reduced when compared with the gold-standard BVAS definition. As expected, encounters incorrectly labelled as relapses by our algorithm tended to be borderline or minor cases, where there was also a degree of clinical uncertainty.

Many limitations related to the requirement for pragmatic study design decisions have already been discussed, including the inclusion of non-standard biomarkers and the assumptions made in creating DE5: IS response. Furthermore, the level of data supporting key definitions and the choice of DEs ranged greatly, with some based on expert opinion, elicited in a structured fashion. The results may have differed if another set of experts were involved and/or an alternative elicitation process was used. Nonetheless, these data are still valid for the method and choices made. Our genetically homogeneous Irish cohort is derived from a universal health system and most had renal involvement. The upper estimate for prevalent patients is approximately 1300.^44 45^ This registry-based study, therefore, equates to about 40% case ascertainment, which may introduce selection bias. However, the baseline characteristics of included patients are similar to international renal cohorts, suggesting the CP will be generalisable to this group. Validation, and potentially recalibration, of the CP in other populations (eg, non-renal cohorts) is paramount before universal adoption. It is plausible that changes in treatment over time, for example, the spread of rituximab use, may affect the CP performance.

Missingness, often ‘not-at-random’, is ubiquitous in observational research and there is no standardised methodology for handling this problem.^14^ Complete-case analysis, as used here, is the most common practice in longitudinal research.^46^ In our study, missingness was most problematic in ‘ANCA level’, particularly in those adjudicated as ‘no relapse’. ESKD was over-represented in these excluded incomplete cases in whom ANCA testing is performed less frequently. These patients are also less likely to attend specialist vasculitis clinics so the frequency of these encounters, requiring adjudication, is limited.

Crucially, the development, validation and deployment of a CP is not a one-off process. It is dynamic, with iterations necessary as new data (eg, biomarkers) and/or the way in which this data is measured or collected arises.^47^ CP performance also deteriorates over time due to natural shifts in epidemiology and the evolution of treatment and care pathways.^14^ A ‘living’ CP would create a paradigm akin to an audit cycle for data quality, to ensure adequate predictive performance is maintained. Embedding of the CP within a knowledge graph, a semantic web-based model for representing interconnected data,^48^ would enhance interoperability across sites, where underlying schemas differ.^49^

This CP for identifying relapse retrospectively demonstrates strong performance using objective, readily accessible registry data. Our electronic algorithm can be used by researchers to calculate the individualised probability of relapse, hence ensuring more accurate outcome ascertainment in real-world research in AAV, where BVAS may be incomplete or inaccurate. In addition to our web application, the algorithm could be directly imbedded into a registry, potentially using a knowledge graph approach, thereby enabling flexible selection of the optimal model, depending on data availability. The tolerance for what is deemed an ‘acceptable’ model and the trade-off between performance metrics can be fine-tuned, depending on the proposed use. This framework could serve as an exemplar for other relapsing-remitting diseases and for automating the identification of other key outcomes or cohorts in registry data.

## Data Availability

Data are available on reasonable request. We would invite any potential research collaborations or data requests through the corresponding author, MAL (mlittle@tcd.ie), on reasonable request, as agreed by participants in their written informed consent (detailed on page 3: https://www.tcd.ie/medicine/thkc/assets/pdf/RKD-Vasculitis-Patient-PIL-ICF-Version-5-07AUG19.pdf). Requests will be considered on a case-by-case basis.
