# Supplementary material for: Computable phenotype for real-world, data-driven retrospective identification of relapse in ANCA-associated vasculitis
Source: RMD Open. 2024 Apr 30;10(2):e003962. doi: 10.1136/rmdopen-2023-003962 (PMC11086371; doi:10.1136/rmdopen-2023-003962)
Supplement: Supplementary data [file rmdopen-2023-003962supp001.pdf]

Supplementary Material

Table of Contents

***Supplementary Material*......1**

Supplementary Methods 1

Data description......1

Steps in building the CP for relapse 2

Patient and Public Involvement 2

Supplementary Figures 4

Supplementary Figure 1: Flowchart demonstrating inclusion and exclusion criteria to determine participants with ANCA-associated vasculitis included in this cohort study......4

Supplementary Figure 2: A). A bar chart demonstrating the percentage of completeness for BVAS across six European vasculitis registries in the FAIRVASC consortium. B). The performance metrics of BVAS when compared to the adjudicated probability of relapse in the Rare Kidney Disease (RKD) registry. C). A stacked bar chart demonstrating the distribution of the recorded BVAS compared to the adjudicated probability of relapse. 5

Supplementary Figure 3: The proportion of patients within each of the five data elements in the model, stratified by the adjudicated probability of relapse......6

Supplementary Figure 4: Receiver operating characteristic (ROC) curve of the complete five-variable model. 7

Supplementary Figure 5: a). Calibration plot of the complete five-variable model. b). a histogram of predicted probabilities......8

Supplementary Figure 6: A dot plot demonstrating the performance of the complete 5-variable model against the 4-level ground truth (adjudicated probability of relapse)......9

Supplementary Figure 7: A dot plot demonstrating the performance of the complete 5-variable stratified by major or minor relapse. 10

Supplementary Figure 8: The performance of a second internal validation......11

Supplemetary Tables 12

Supplementary Table 1: Cross-tabulation of the ground truth label (adjudicated probability of relapse) versus the predicted relapse label......12

Supplementary Table 2: Subgroup analysis: the performance metrics of the complete 5-variable multi-level logistic regression model applied to those with and without kidney involvement. 13

Supplementary Table 3: The mean (95% CI) of all performance metrics for the 31 model combinations of the 5 data elements, ranked by F1-Score......14

Supplementary Table 4: Data elements currently recorded in registries across the FAIRVASC initiative and the model EUVAS registry......16

Supplementary Methods

Data description

Patient encounters were usually recorded at three-monthly clinic intervals and at the time of potential relapse, with a targeted annual minimum. The observation period was from the date of diagnosis to the last documented visit or death. Given the observational nature of the study, patient management was at the discretion of their clinician. Data collection was in agreement with the core dataset and interoperability principles outlined by EUVAS. A sample size estimation was not possible given the novelty of this study.

Scott J, *et al. RMD Open* 2024; 10:e003962. doi: 10.1136/rmdopen-2023-003962

Steps in building the CP for relapse

Step 1 - Independent expert adjudication of encounters to assign the reference probability of relapse (ground truth)

Remission was defined as the absence of symptoms, signs and/or objective evidence of vasculitis activity. Encounters were adjudicated (in advance of this study) by a committee of expert clinicians (at least 2 of: JS, SM, NC and ML), using the patient's entire medical records. The medical records included clinical notes, medication records and all laboratory, radiological and histopathological data across the patient's entire longitudinal disease course. In keeping with clinical practice, and the observational nature of the study, some investigations were not performed, or the results were missing in a small number. The committee assigned one of four probabilities categories: definite, high probable, possible or no relapse. The degree of certainty corresponded to the strength of supporting objective evidence: if clear histopathological evidence of active vasculitis was present a 'definite relapse' label was applied, while suggestive laboratory or radiological evidence portended to a 'high probability of relapse' and encounters with a convincing clinical scenario but lacking objective evidence were labelled as 'possible relapse'.

Step 2 - Selection of data elements and corresponding value sets.

Models with a small number of predictors are more attractive to implement in the clinical arena (minimising data requirements and maximising understandability). Limiting complexity also reduces the margin for error when applying the model to other datasets. Rather than predicting future relapse, the aim was to select data elements that uniformly characterise patient encounters, thereby objectively aiding retrospective relapse labelling, and allowing automation of the expert adjudication process.

Expert domain knowledge was elicited by a semi-formalised Delphi approach, to inform the selection of data elements. A methodology paper is currently being prepared for publication by our group. The most informative biomarkers with regards to relapse were: anti-MPO, anti-PR3, creatinine, uCD163, CRP, proteinuria (negative to positive) and haematuria (negative to positive).

Patient and Public Involvement

Patient involvement was primarily through the national patient group, 'Vasculitis Ireland Awareness', focus groups and 'question and answer' sessions at their national annual meeting. Patients were instrumental in prioritising relapse as a key focus for research, which led to this study. AAV relapse is a recognised target of research for the wider vasculitis community also. Patients were involved in the design and conduct of this research. Julie Power, a patient representative, joined the study steering committee. We developed a study newsletter in conjunction with VIA (hosted on their website: https://vasculitis-ia.org/) to inform participants and the wider vasculitis community about study updates. We will disseminate study findings

through this website, social media channels and at patient sessions at the biannual International Vasculitis and ANCA Workshop.

Supplementary Figures

Supplementary Figure 1: Flowchart demonstrating inclusion and exclusion criteria to determine participants with ANCA-associated vasculitis included in this cohort study.

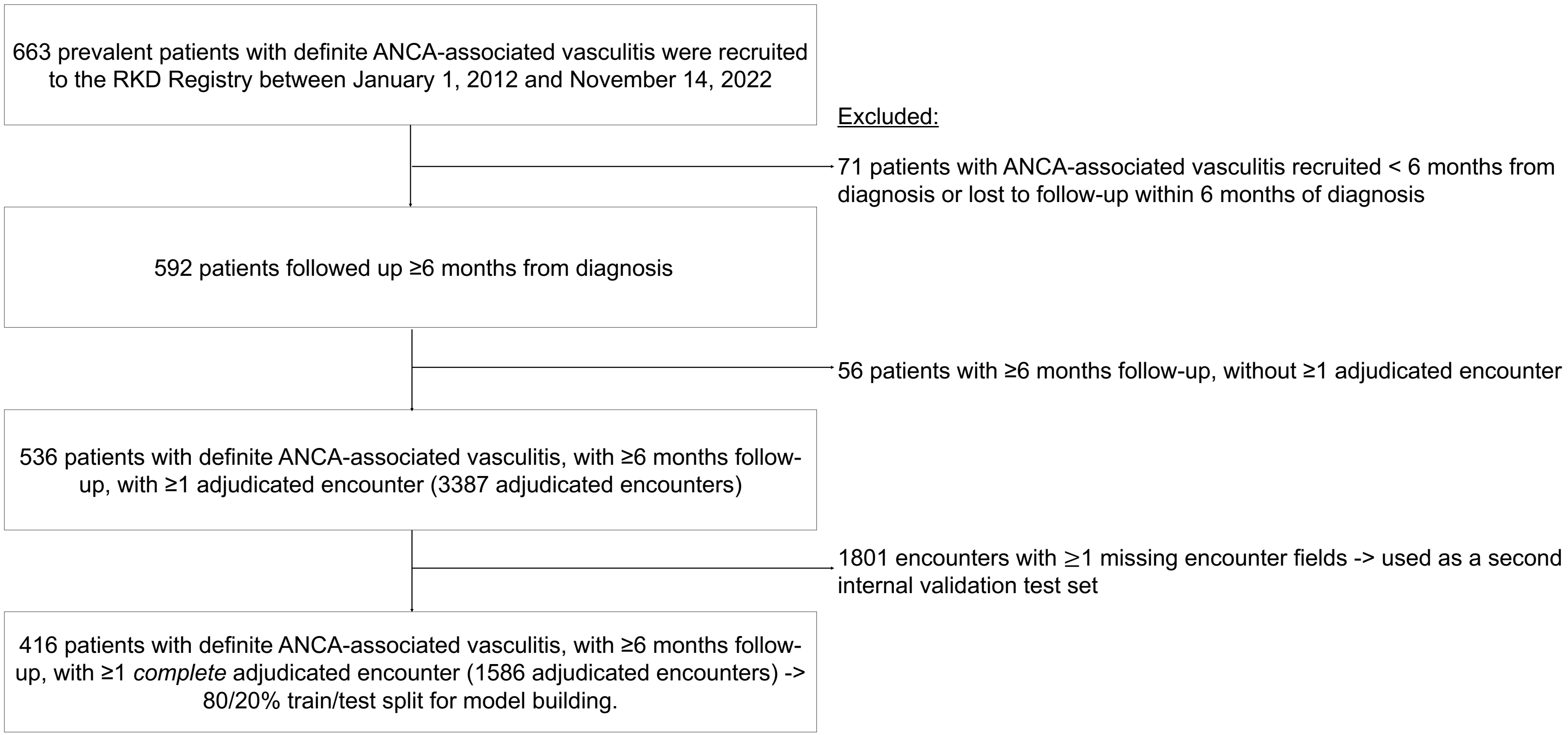

Supplementary Figure 2: A). A bar chart demonstrating the percentage of completeness for BVAS across six European vasculitis registries in the FAIRVASC consortium (Italian, The joint Vasculitis Registry in German-speaking countries (Germany/Austria/Switzerland, GeVas), Polish Vasculitis Registry (PolVas), Skane Sweedish Registry, French Vasculitis Study Group (FVSG) and Czech Vasculitis Registry). B). The performance metrics of BVAS when compared to the adjudicated probability of relapse in the Rare Kidney Disease (RKD) registry. C). A stacked bar chart demonstrating the distribution of the recorded BVAS compared to the adjudicated probability of relapse across all encounters with an available BVAS in the RKD registry (31%). The shading of the bars denote the adjudicated probability of relapse as per the legend.

A.

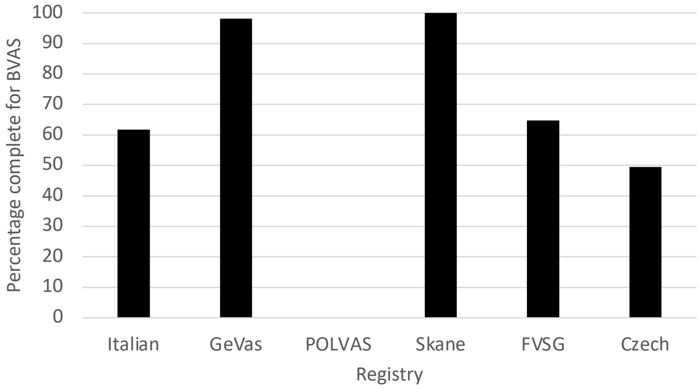

B.

Performance Metric	BVAS >0
F1-Score	0.7013
Sensitivity	0.9748
Specificity	0.8589
Positive predictive value	0.5477
Negative predictive value	0.9949
Accuracy	0.8762
Prevalence of relapse	0.15

C.

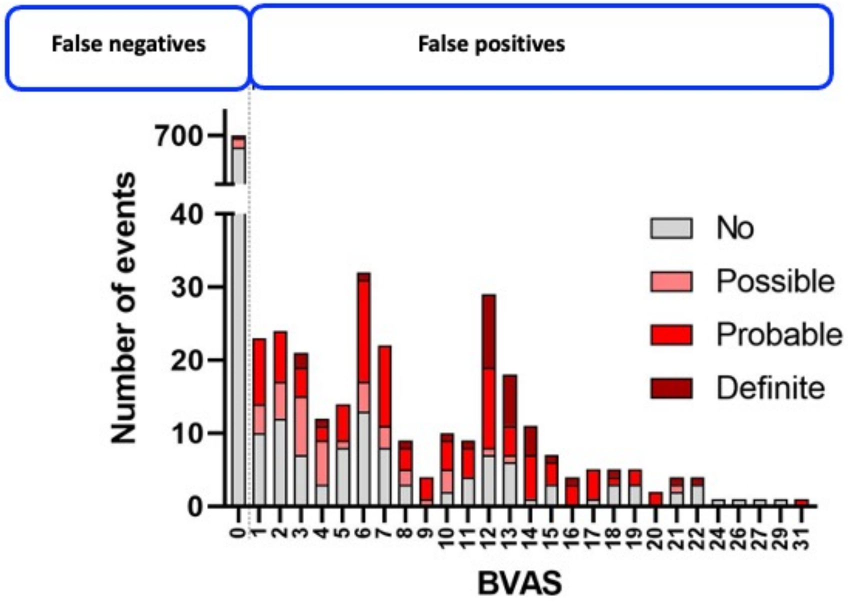

5

Supplementary Figure 3: The proportion of patients within each of the five data elements in the model, stratified by the adjudicated probability of relapse

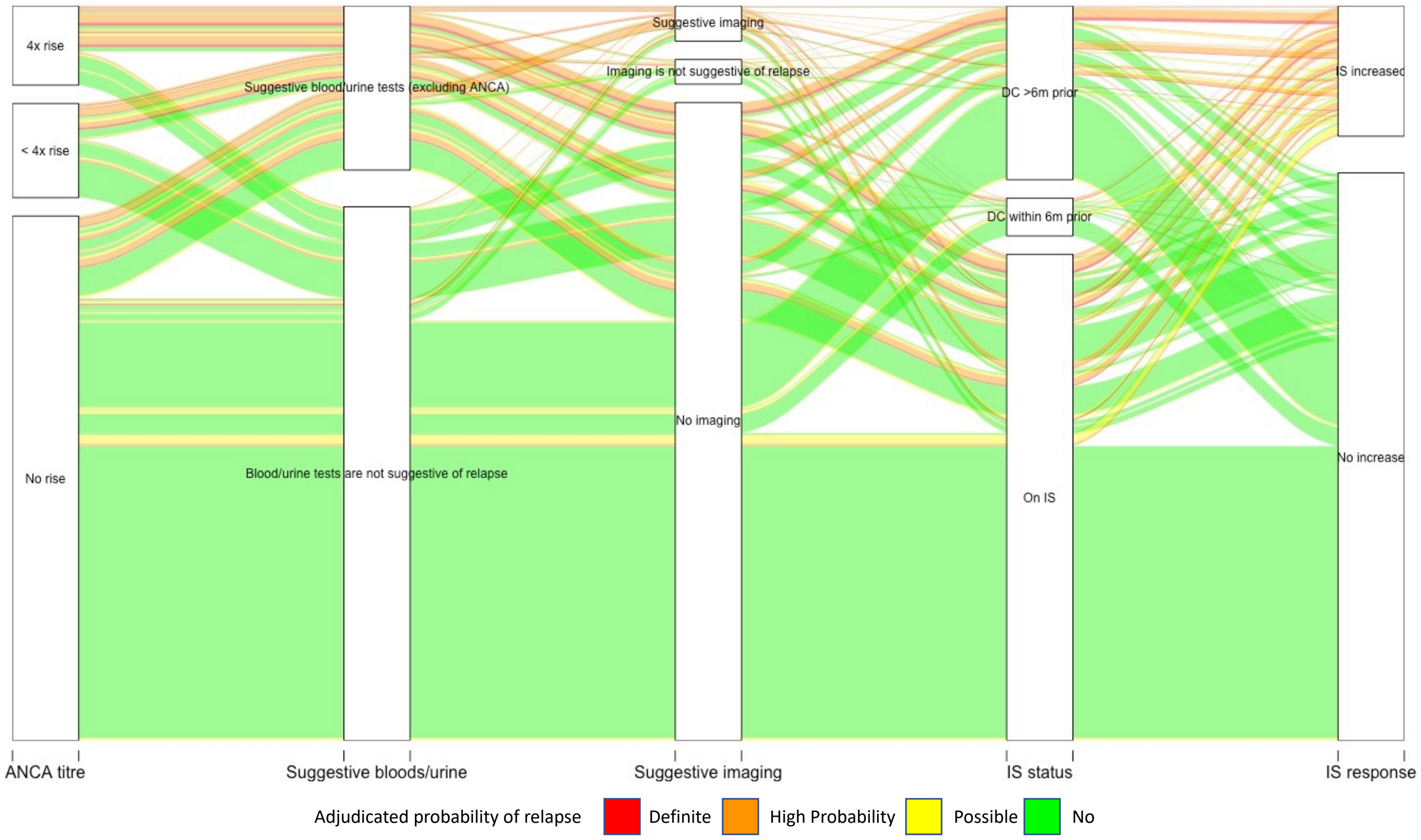

Supplementary Figure 4: Receiver operating characteristic (ROC) curve of the complete five-variable model. The ROC curve is determined by plotting the false positive rate (1- specificity) against the true positive rate (sensitivity). The area under the curve (AUC) is an aggregate measure of the ability of the model to distinguish between classes. Importantly, it is scale- and classification-threshold invariant. It is also agnostic to the prevalence of the outcome. For these reasons, the precision-recall curve is more useful to assess the accuracy of the model in our imbalanced dataset.

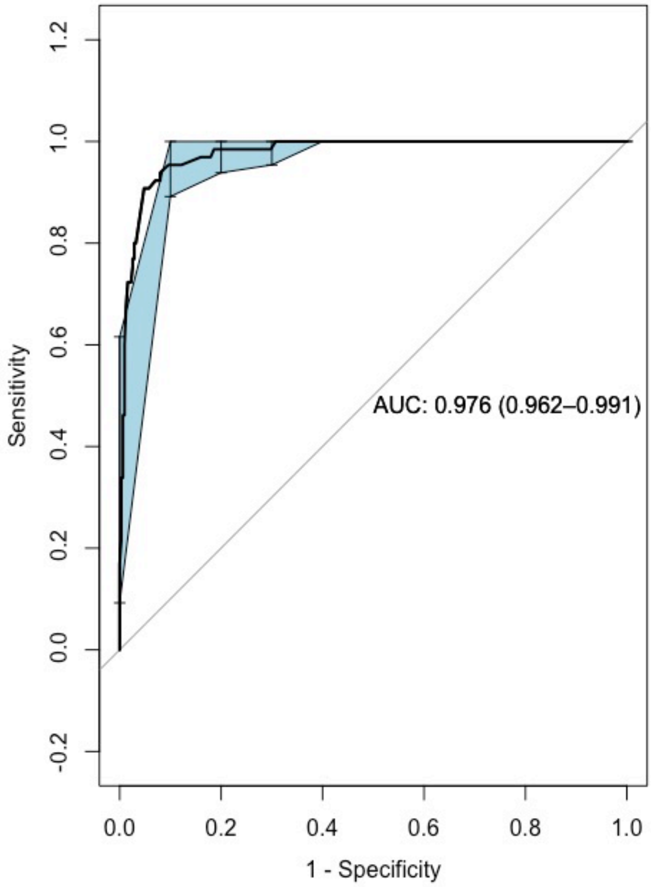

Supplementary Figure 5: a). Calibration plot of the complete five-variable model. The x-axis represents the midpoint of the fitted predicted probability of relapse of each bin (n=11), while the y-axis represents the observed proportion of relapse. The relatively narrow confidence intervals crossing the diagonal line suggest the model is well calibrated at the extremes (predicted probability 0-10% and 90-100%), but the model is underpowered in between. The predominance of cases at the extremes and lack of cases with a probability between 0.1-0.9 is demonstrated in b). a histogram of predicted probabilities.

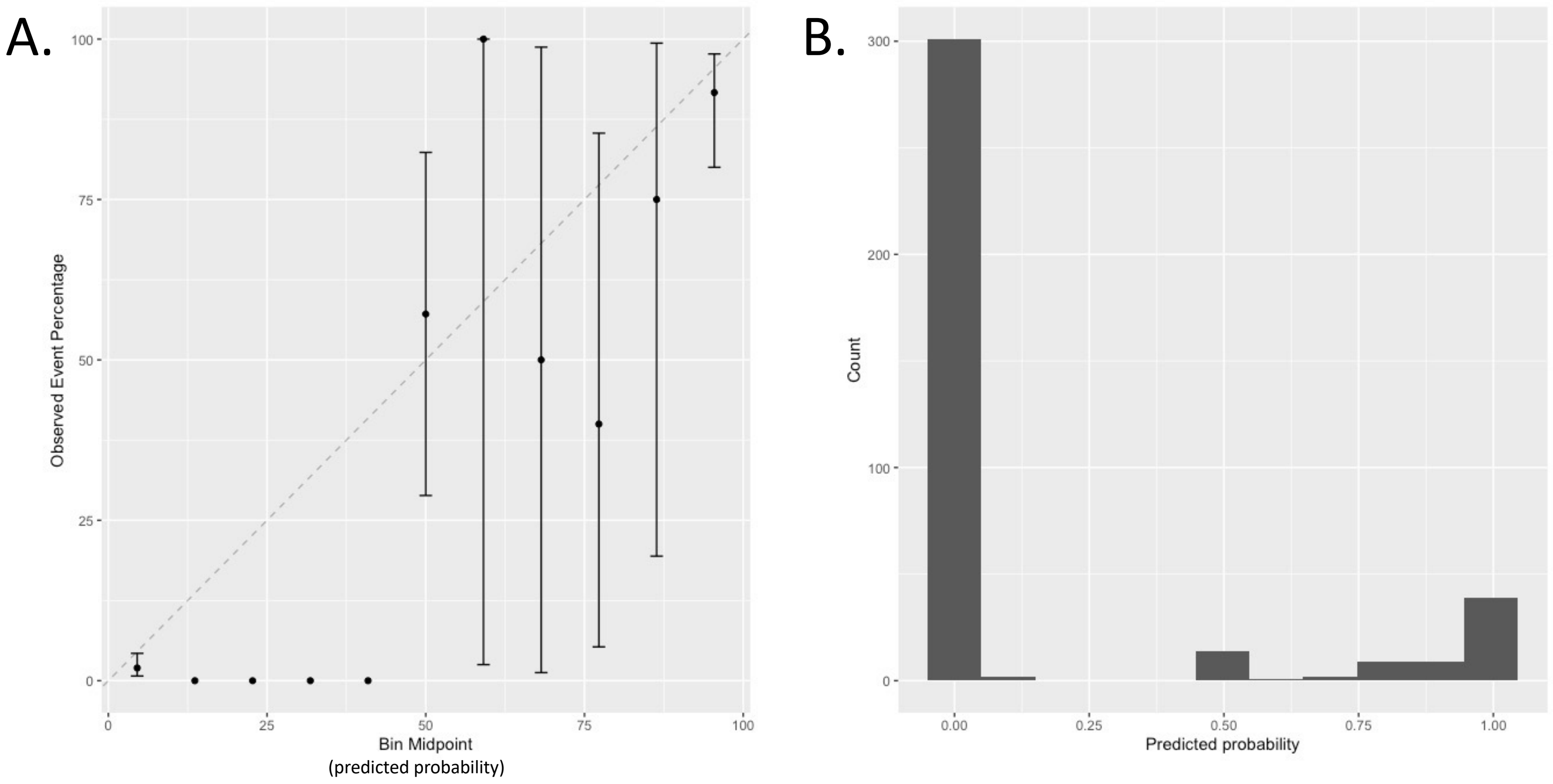

Supplementary Figure 6: A dot plot demonstrating the performance of the complete 5-variable model against the 4-level ground truth (adjudicated probability of relapse). The dotted line denotes the optimal cut-point (0.48), derived by maximizing the F1-Score. Above this line predicted probabilities are dichotomized into relapse=1, while below the line relapse=0.

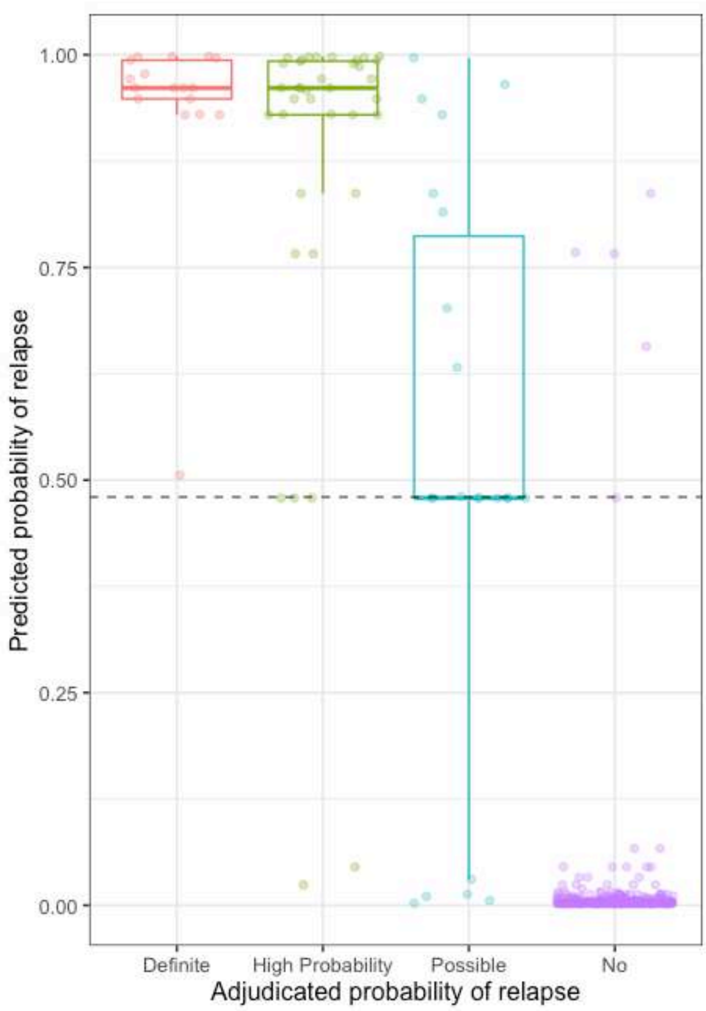

***False negative* examples** (where ‘Adjudicated probability of relapse’ = ‘High Probability’ or ‘Possible’, but predicted probability of relapse by the model is <0.48 (i.e. no relapse): instances where disease activity was increased but there was no escalation in immunosuppression (IS), such as in cases of renal relapse resulting in end-stage kidney disease, where the risk of IS outweighed the benefit. Similarly, instances of low-grade disease activity (non-organ/life-threatening), where a ‘watch and wait’ approach was employed; these are relapses, but untreated at that encounter. In these scenarios the model would underestimate relapse. ***False positive* example** (where ‘Adjudicated probability of relapse’ = ‘No’, but predicted probability of relapse by the model is >0.48 (i.e. relapse): IS increased by physician, but on retrospective review encounter deemed a relapse mimic.

Supplementary Figure 7: A dot plot demonstrating the performance of the complete 5-variable stratified by major or minor relapse. The dotted line denotes the optimal cut-point (0.48), derived by maximizing the F1-Score. Above this line predicted probabilities are dichotomized into relapse=1, while below the line relapse=0. There is a greater degree of uncertainty of the model with minor relapses, compared to major relapses.

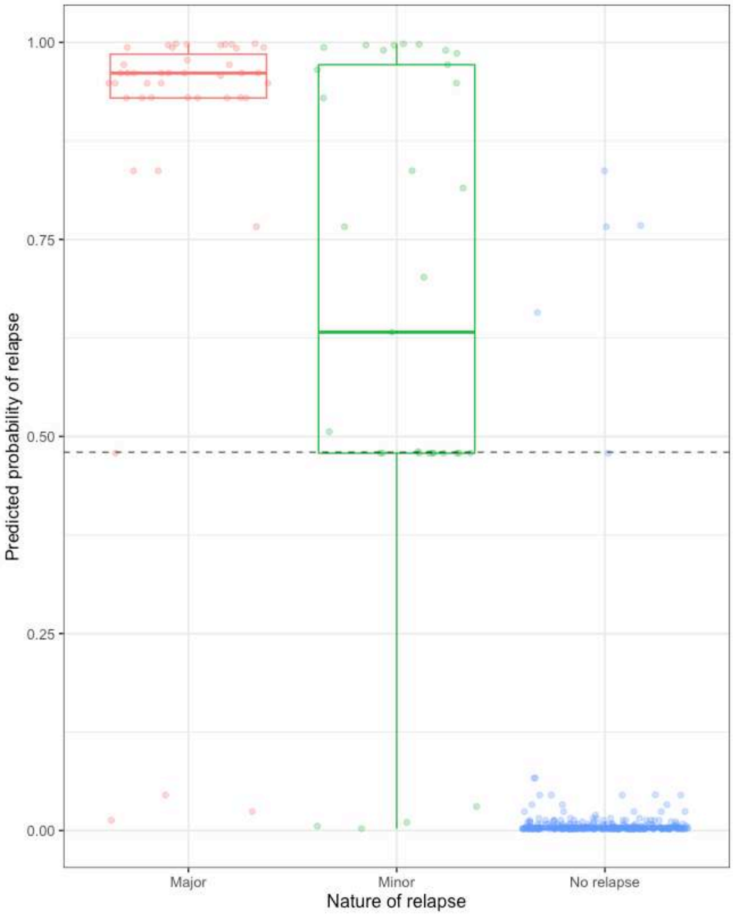

Supplementary Figure 8: Applying the web interface, a second internal validation was performed using the 1801 incomplete encounters (initially excluded due to missingness of data elements). The performance of the model remained high.

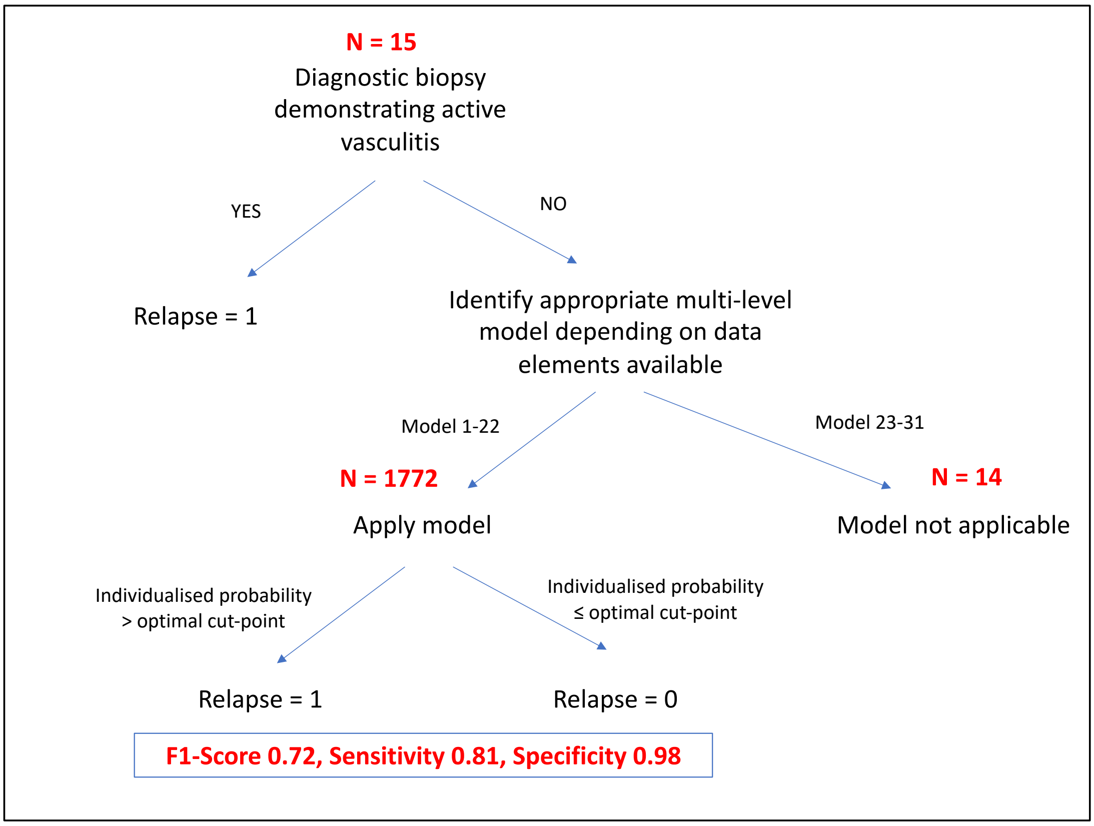

Of the 1801 incomplete encounters, 15 had diagnostic histopathology and hence were labelled as relapse. No individualised probability was returned for 14 observations as their variable missingness was too high, corresponding to models 23-31. The prevalence of relapse in this cohort was much lower (4.1%), presumably because completion of data fields was higher in cases where relapse was suspected. For this validation, the F1-Score point estimate (0.72) was still higher than BVAS (0.7), while sensitivity was lower (0.81) and specificity remained very high (0.98).

Supplemetary Tables

Supplementary Table 1: Cross-tabulation of the ground truth label (adjudicated probability of relapse) versus the predicted relapse label from the complete 5-variable multi-level model described in Table 3, whereby 0=no relapse, 1=relapse.

	Ground truth	
Prediction	0	1
0	297	6
1	15	59

Scott J, *et al. RMD Open* 2024; 10:e003962. doi: 10.1136/rmdopen-2023-003962

Supplementary Table 2: Subgroup analysis: the performance metrics of the complete 5-variable multi-level logistic regression model applied to those with and without kidney involvement.

Performance Metric	Kidney involvement (N=280)	No kidney involvement (N=97)
F1-Score	0.8679	0.7879
Sensitivity	0.9388	0.8125
Specificity	0.9524	0.9506
Positive predictive value	0.8070	0.7647
Negative predictive value	0.9865	0.9625
Accuracy	0.9500	0.9278
Area under ROC curve	0.9849	0.9541
Prevalence of relapse	0.1750	0.1649

Supplementary Table 3: The mean (95% CI) of all performance metrics for the 31 model combinations of the 5 data elements, ranked by F1-Score. Models 1-22 have a classification accuracy at least as good as the BVAS >0 definition of relapse, denoted by the 95% CI of their F1-score crossing the F1-score point estimate (0.7) for the BVAS >0 definition.

Model Rank	Data elements	Optimal cut point (max F1-S)	F1-score	Sensitivity	Specificity	PPV	NPV	Accuracy	AUC
1	2,3,4,5	0.4363	0.8507 (0.7745-0.9269)	0.8909 (0.8000-0.9819)	0.9589 (0.9300-0.9878)	0.8166 (0.7065-0.9267)	0.9774 (0.9584-0.9965)	0.9474 (0.9191-0.9757)	0.9792
2	1,3,4,5	0.456	0.8490 (0.7728-0.9252)	0.8947 (0.8052-0.9842)	0.9571 (0.9305-0.9838)	0.8098 (0.7059-0.9138)	0.9781 (0.9588-0.9973)	0.9465 (0.9187-0.9743)	0.9615
3	1,2,3,4,5	0.3684	0.8479 (0.7726-0.9233)	0.8949 (0.7972-0.9925)	0.9564 (0.9255-0.9874)	0.8088 (0.6950-0.9227)	0.9782 (0.9578-0.9985)	0.9460 (0.9178-0.9742)	0.9763
4	2,4,5	0.5822	0.8460 (0.7667-0.9252)	0.8836 (0.7945-0.9726)	0.9588 (0.9327-0.9848)	0.8134 (0.7075-0.9193)	0.9757 (0.9558-0.9957)	0.9459 (0.9170-0.9748)	0.9703
5	1,2,3,5	0.3789	0.8457 (0.7700-0.9213)	0.8922 (0.7835-1.0010)	0.9561 (0.9244-0.9877)	0.8074 (0.6931-0.9216)	0.9777 (0.9554-1.0000)	0.9453 (0.9174-0.9731)	0.9751
6	1,2,4,5	0.4759	0.8453 (0.7686-0.9219)	0.8875 (0.7908-0.9841)	0.9571 (0.9248-0.9894)	0.8102 (0.6928-0.9276)	0.9767 (0.9565-0.9968)	0.9453 (0.9162-0.9744)	0.9701
7	1,4,5	0.693	0.8448 (0.7660-0.9236)	0.8813 (0.7882-0.9745)	0.9588 (0.9349-0.9827)	0.8130 (0.7128-0.9132)	0.9753 (0.9548-0.9959)	0.9456 (0.9176-0.9736)	0.9452
8	2,3,5	0.423	0.8447 (0.7700-0.9195)	0.8950 (0.7960-0.9939)	0.9547 (0.9228-0.9867)	0.8032 (0.6877-0.9186)	0.9782 (0.9582-0.9983)	0.9447 (0.9165-0.9728)	0.978
9	3,4,5	0.567	0.8425 (0.7616-0.9234)	0.8902 (0.8013-0.9792)	0.9551 (0.9268-0.9835)	0.8019 (0.6890-0.9147)	0.9771 (0.9574-0.9967)	0.9440 (0.9146-0.9735)	0.9609
10	1,3,5	0.4992	0.8425 (0.7655-0.9195)	0.8982 (0.8007-0.9958)	0.9530 (0.9263-0.9797)	0.7956 (0.6908-0.9003)	0.9786 (0.9573-1.0000)	0.9436 (0.9158-0.9714)	0.9552
11	1,2,5	0.4954	0.8415 (0.7637-0.9192)	0.8939 (0.7951-0.9926)	0.9533 (0.9187-0.9880)	0.7985 (0.6752-0.9219)	0.9779 (0.9574-0.9984)	0.9432 (0.9132-0.9733)	0.9658
12	4,5	0.7437	0.8399 (0.7580-0.9218)	0.8855 (0.7891-0.9820)	0.9552 (0.9284-0.9819)	0.8009 (0.6925-0.9093)	0.9761 (0.9548-0.9974)	0.9433 (0.9138-0.9727)	0.9339
13	3,5	0.765	0.8392 (0.7597-0.9188)	0.8911 (0.7946-0.9875)	0.9533 (0.9268-0.9799)	0.7953 (0.6896-0.9010)	0.9772 (0.9561-0.9983)	0.9427 (0.9142-0.9712)	0.9541
14	1,5	0.7764	0.8386 (0.7585-0.9188)	0.8888 (0.7898-0.9879)	0.9537 (0.9265-0.9810)	0.7963 (0.6873-0.9053)	0.9767 (0.9547-0.9987)	0.9426 (0.9138-0.9713)	0.9373
15	2,5	0.6594	0.8386 (0.7585-0.9188)	0.8888 (0.7898-0.9879)	0.9537 (0.9265-0.9810)	0.7963 (0.6873-0.9053)	0.9767 (0.9547-0.9987)	0.9426 (0.9138-0.9713)	0.9649
16	5	0.8599	0.8386 (0.7585-0.9188)	0.8888 (0.7898-0.9879)	0.9537 (0.9265-0.9810)	0.7963 (0.6873-0.9053)	0.9767 (0.9547-0.9987)	0.9426 (0.9138-0.9713)	0.9237

Model Rank	Data elements	Optimal cut point (max F1-S)	F1-score	Sensitivity	Specificity	PPV	NPV	Accuracy	AUC
17	1,2,3,4	0.3618	0.6708 (0.5775-0.7641)	0.7050 (0.5514-0.8586)	0.9181 (0.8402-0.9961)	0.6534 (0.4735-0.8332)	0.9394 (0.9120-0.9668)	0.8826 (0.8303-0.9348)	0.8827
18	1,2,3	0.4017	0.6678 (0.5704-0.7652)	0.6938 (0.5306-0.8570)	0.9214 (0.8385-1.0044)	0.6609 (0.4646-0.8572)	0.9371 (0.9039-0.9702)	0.8828 (0.8290-0.9367)	0.8878
19	2,3,4	0.2386	0.6431 (0.5615-0.7247)	0.8150 (0.7120-0.9181)	0.8538 (0.8003-0.9072)	0.5332 (0.4375-0.6288)	0.9580 (0.9351-0.9808)	0.8474 (0.8034-0.8914)	0.8632
20	2,3	0.3157	0.6397 (0.5580-0.7214)	0.8139 (0.7227-0.9051)	0.8518 (0.8017-0.9019)	0.5292 (0.4292-0.6291)	0.9574 (0.9348-0.9800)	0.8454 (0.8029-0.8879)	0.8675
21	1,2,4	0.3749	0.6179 (0.5288-0.7070)	0.6764 (0.5040-0.8489)	0.8952 (0.8048-0.9856)	0.5887 (0.3759-0.8015)	0.9319 (0.8971-0.9667)	0.8581 (0.7988-0.9174)	0.8597
22	1,2	0.4303	0.6146 (0.5264-0.7027)	0.6826 (0.4770-0.8881)	0.8900 (0.7954-0.9845)	0.5808 (0.3761-0.7854)	0.9333 (0.8964-0.9701)	0.8553 (0.7954-0.9151)	0.8629
23	2	0.54	0.6095 (0.5221-0.6970)	0.7285 (0.6180-0.8391)	0.8659 (0.8175-0.9143)	0.5265 (0.4233-0.6297)	0.9398 (0.9107-0.9690)	0.8426 (0.7977-0.8874)	0.8196
24	2,4	0.4966	0.6095 (0.5221-0.6970)	0.7285 (0.6180-0.8391)	0.8659 (0.8175-0.9143)	0.5265 (0.4233-0.6297)	0.9398 (0.9107-0.9690)	0.8426 (0.7977-0.8874)	0.8139
25	1,3,4	0.3037	0.5545 (0.4635-0.6455)	0.6192 (0.4214-0.8170)	0.8744 (0.7615-0.9873)	0.5203 (0.3551-0.6854)	0.9199 (0.8870-0.9527)	0.8318 (0.7605-0.9031)	0.8185
26	1,3	0.3224	0.5470 (0.4477-0.6464)	0.6383 (0.4471-0.8295)	0.8563 (0.7297-0.9828)	0.4963 (0.3165-0.6761)	0.9222 (0.8918-0.9525)	0.8200 (0.7363-0.9037)	0.823
27	1,4	0.3045	0.4751 (0.3845-0.5656)	0.5601 (0.4106-0.7096)	0.8386 (0.7633-0.9138)	0.4189 (0.3071-0.5307)	0.9039 (0.8687-0.9391)	0.7916 (0.7335-0.8498)	0.7438
28	1	0.3148	0.4714 (0.3819-0.5608)	0.5764 (0.4391-0.7137)	0.8248 (0.7702-0.8793)	0.4019 (0.3110-0.4928)	0.9056 (0.8701-0.9411)	0.7828 (0.7361-0.8296)	0.7366
29	3,4	0.2981	0.4104 (0.2953-0.5256)	0.3902 (0.1735-0.6069)	0.8845 (0.6379-1.0000)	0.5393 (0.1336-0.9451)	0.8779 (0.8543-0.9014)	0.8019 (0.6282-0.9756)	0.7017
30	3	0.3635	0.3965 (0.2594-0.5337)	0.4080 (0.0000-0.8996)	0.8267 (0.1524-1.0000)	0.6248 (0.3987-0.8509)	0.8738 (0.8473-0.9004)	0.7573 (0.2807-1.0000)	0.6605
31	4	0.1589	0.2966 (0.2408-0.3524)	0.7948 (0.2391-1.0000)	0.2617 (0.0000-0.9684)	0.2399 (0.1896-0.2901)	0.8651 (0.8438-0.8864)	0.3532 (0.0000-0.8477)	0.551
BVAS			0.7013	0.9748	0.8589	0.5477	0.9949	0.8762	

The model rank and F1-Scores correspond to that in Figure 3. Prevalence of relapse 17% in models 1-31 and 15% in the BVAS analysis. Data element (DE) key: 1=ANCA titre, 2=Suggestive bloods/urine, 3=Suggestive imaging, 4=Immunosuppressive (IS) status, 5=IS response. 95% confidence interval (95% CI), Birmingham vasculitis activity score (BVAS), positive predictive value (PPV), negative predictive value (NPV), area under the receiver operating curve (AUC).

Supplementary Table 4: Data elements currently recorded in registries across the FAIRVASC initiative and the model EUVAS registry

	Registry					
**Data Element**	FVSG	GeVas	Skane	Czech	PolVas	Model EUVAS
ANCA titre*	■	■		■		■
*Suggestive bloods/urine***						■
Creatinine	■	■		■		■
Haematuria	■	■		■		■
Proteinuria	■	■		■		■
C-Reactive Protein	■	■		■		■
urine soluble CD163						
Suggestive imaging		■				■
IS Status	■	■	■	■		■
IS response***	■	■		■		■
Diagnostic biopsy	■	■		■		■

*ANCA titre is only positive/negative for GEVAS
**Change in creatinine/haematuria/proteinuria are scored as per BVAS in FVSG registry
**Haematuria/proteinuria are scored as per BVAS in Czech registry
***IS response inferred from medication variables for FVSG and Czech registries.
Of note, UKIVAS is also a partner of the FAIRVASC initiative but does not record follow-up encounter data.
French Vasculitis Study Group (FVSG), The joint Vasculitis Registry in German-speaking countries (Germany/Austria/Switzerland, GeVas), Skane Sweedish Registry, Czech Vasculitis Registry, Polish Vasculitis Registry (PolVas), UK and Ireland Vasculitis Society (UKIVAS), European Vasculitis Society (EUVAS).
